# Work engagement, job satisfaction, and turnover intentions among family medicine residency program managers

**DOI:** 10.5116/ijme.5e3e.7f16

**Published:** 2020-02-28

**Authors:** Samuel Ofei-Dodoo, Myra Chantel Long, Morgan Bretches, Bobbi J. Kruse, Cheryl Haynes, Caren Bachman

**Affiliations:** 1 Family and Community Medicine, University of Kansas School of Medicine-Wichita, USA; 2 University of Minnesota Medical Center Family Medicine Residency Program, Minneapolis, USA; 3 The Southern Regional Area Health Education Center Family Medicine Residency, Fayetteville, NC, USA; 4 Smoky Hill Family Medicine Residency Program of the University of Kansas School of Medicine-Salina, USA

**Keywords:** Work engagement, job satisfaction, turnover intentions, family medicine, residency program managers

## Abstract

**Objectives:**

The authors examined the associations between work engagement, job satisfaction, and turnover intentions among family medicine residency (FMR) managers.

**Methods:**

We conducted a cross-sectional online survey of 511 FMR manager members of the Association of Family Medicine Administration using purposive sampling. The Utrecht Work Engagement Scale, Job Satisfaction Survey, and Boshoff and Allen’s 3-item scale were used to assess work engagement, job satisfaction, and turnover intentions respectively. Descriptive statistics, Chi-Square tests, Pearson’s correlations, 2-way contingency table analysis, and hierarchical regression analyses were used to analyze the data.

**Results:**

The response rate was 70.6% (389/551). Work engagement was positively correlated with job satisfaction (r_[387]_=.513, p<.001) and negatively correlated with turnover intentions (r_[368]_=.580, p<.001). Turnover intention was negatively correlated with job satisfaction (r_[387]_=-.690, p<.001). Positive assessment of nature of work (t_[364]_=15.06, p<.001), fringe benefits (t_[364]_=6.89, p<.001), communication (t_[364]_=2.27, p<.05), and promotion (t_[364]_=2.48, p<.05) predicted work engagement. Work engagement (t_[364]_=-4.31, p<.001), pay (t_[364]_=-3.71, p<.001), supervision (t_[364]_=-3.51, P<.01), contingent rewards (t_[364]_=-2.39, p<.05), nature of work (t_[364]_=-2.16, p<.05), and communication (t_[364]_=-2.15, p<.05) predicted turnover intentions.

**Conclusions:**

Our findings demonstrate associations between work engagement, job satisfaction, and turnover intentions. When medical residency managers are emotionally and cognitively engaged at work, they tend to remain in the organization, validating and rewarding organizations that foster employee engagement. Further studies are needed to establish a causal relationship between work engagement, job satisfaction, and turnover intentions and to investigate other potential factors that could contribute to enriching the job satisfaction of this crucial group of professionals.

## Introduction

Family medicine residency (FMR) managers are crucial to the success of family medicine residency programs. Historically, the role of the residency manager has been administrative and clerical; however, since the Accreditation Council for Graduate Medical Education’s (ACGME) Next Accreditation System (NAS) began in 2013 in the United States of America, managers’ roles have expanded to include more managerial and liaison responsibilities. In addition to a significant increase in data management and accreditation validation documentation responsibilities, managers now commonly oversee communications between program directors and faculty members, residents, applicants, staff and representatives of hospitals, medical schools, and multiple external organizations.^1^^-^^3^ Residency program managers insofar are responsible for the administrative duties in medical residency programs within a teaching hospital or medical facility. A wide variety of job titles are used for FMR managers, including program coordinator, academic coordinator, program administrator, residency manager, and program manager. This study uses the title of “family medicine residency manager” to encompass all the various job titles of FMR administrators.

Work engagement, defined as “a positive, fulfilling work-related state of mind that is characterized by vigor, dedication, and absorption,”^4^ has been validated as one of the most important factors in the success of any work environment.^5^ Work engagement is associated with positive work outcomes such as low turnover intentions,^6^ reduced absenteeism,^7^ increased productivity,^8^ and job satisfaction in diverse groups including hospitality workers,^8^^,^^9^ telecommunication workers,^6^ and medical residents.^10^^,^^11^ We were unable to identify any published work engagement studies involving FMR managers.

Job satisfaction defined as “the pleasure people derive from their work, including their ability to positively affect the lives of people through work”^2^ has been studied using  nine  general domains (satisfaction with pay, opportunities for promotion, fringe benefits, contingent rewards, nature of supervision, relationships with co-workers, nature of work, communications, and working conditions).^12^

Attitudinal work outcomes, job satisfaction and turnover intentions have predominantly been studied in relation to the negative outcome of burnout,^13^^-^^16^ but not to the positive outcome of enhanced work engagement, experientially among FMR managers. Job turnover intention is defined as “a conscious and deliberate willingness to leave an organization.”^17^ Although no standard framework has been developed to understand why employees leave voluntarily, factors such as poor workplace communication, hostile work environment, unexplained work task, inequitable salary and benefits packages, have been implicated.^18^^,^^19^ A 2018 study reported that 51% of 307 FMR managers had been employed in their current organization for less than five years, suggesting a high turnover rate.^2^ There is currently no published data on FMR managers’ turnover intentions. Studies in diverse industries have found turnover intentions to be an accurate predictor of actual turnover.^20^^,^^21^ Studies have also consistently shown a strong and inverse association between job satisfaction and employee turnover intentions.^21^^-^^25^

Given the increased volume and significance of job responsibilities and duties expected of FMR managers after the adaptation of NAS, they could be vulnerable to experiencing increased job-related stress and the negative outcomes of decreased work engagement, job satisfaction and increased turnover. We studied job satisfaction and turnover intentions among FMR managers and examined the associations between work engagement, job satisfaction, turnover intentions, and self-reported demographic characteristics. Our specific hypotheses based on the described conceptual framework were:

     1.FMR manager work engagement, job satisfaction, and turnover intentions are related.

     2.Job satisfaction domains (pay, promotion, supervision, fringe benefits, contingent rewards, operating procedures, co-workers, nature of work, and communication) are predictors of work engagement.

     3.FMR manager characteristics (sex, employment status, tenure [length of service in current organization], annual salary, and educational status), job satisfaction domains (pay, promotion, supervision, fringe benefits, contingent rewards, operating procedures, co-workers, nature of work, and communication), and work engagement are predictors of turnover intentions.

## Methods

### Study design and participants

This study was a cross-sectional survey of FMR managers who were members of the Association of Family Medicine Administration (AFMA), which is a professional organization dedicated to the professional growth and development of FMR managers. We used purposive sampling to identify the FMR managers in the United States of America to participate in the study. The study participants completed an anonymous, 59-item online survey that comprised of questions regarding work engagement, job satisfaction, turnover intentions, and demographic information. A sample size of 180 was calculated as necessary for adequate power (> 0.85) to detect a significant association of p < 0.05 among the variables.^26^ The University of Kansas School of Medicine-Wichita Institutional Review Board granted exemption as the study employed survey procedures where information obtained was recorded in such a manner that the identity of the participants could not readily be ascertained, directly or through identifiers linked to the participants. As shown in Table 1, almost all (98.3%) of the participants were female. About half had been in their current position for five years or less (54.9%), reported directly to their family medicine residency program director (51%), or held a bachelor’s or higher degree (52%). Likelihood Ratio Chi-Square tests showed no significant relation between participant gender (male vs female), job satisfaction, and turnover intentions.

### Setting

The study was conducted in the United States of America at the University of Kansas School of Medicine-Wichita campus. The University of Kansas School of Medicine-Wichita is a full four-year community-based school that trains medical students where the majority often choose to go into family medicine specialty.

**Table 1:** Participant characteristics (N = 389)

Variable		Participant n (%)
Sex		
	Male	6 (1.7)
	Female	344 (98.3)
	Missing^*^	39
Employment status		
	Part-time	7 (2.0)
	Full-time	343 (98.0)
	Missing^*^	39
Tenure, years		
	<5	189 (54.9)
	6 - 10	57 (16.6)
	11 - 15	39 (11.3)
	16 - 20	23 (6.7)
	21 - 25	22 (6.4)
	26 - 30	7 (2.0)
	>30	7 (2.0)
	Missing^*^	45
Direct supervisor		
	Residency program director	198 (50.9)
	Clinic manager	34 (8.7)
	Other	157 (40.4)
Annual Salary		
	< $35,000	27 (7.6)
	$35,000 - $44,999	59 (16.7)
	$45,000 - 54,999	117 (33.1)
	$55,000 - $64,999	76 (21.5)
	≥$65,000	75 (21.2)
	Missing^* ^	35
Community location of program		
	Inner-city	54 (15.4)
	Suburban	108 (30.8)
	Rural	112 (31.9)
	Urban	77 (21.9)
	Missing^*^	38
Highest educational Level		
	Graduated from high school	43 (12.3)
	Attended college but did not complete	59 (16.9)
	Completed associate degree (AA, AS, etc.)	66 (18.9)
	Completed bachelor's degree (BA, BS, etc.)	123 (35.1)
	Completed master's degree (MA, MS, etc.)	57 (16.3)
	Completed doctorate degree (MD, JD, PhD, etc.)	2 (0.6)
	Missing^*^	39

*The number of managers who completed the survey but did not provide an answer to this specific question. Missing responses were excluded from the total before percentages were calculated.

### Study instruments

Work engagement: we used the Utrecht Work Engagement Scale (UWES-9), a validated 9-item inventory assessing work engagement across the dimensions of vigor, dedication, and absorption.^4^ In this scale, vigor refers to the energy and mental resilience employees display at work; dedication addresses participants’ sense of significance, enthusiasm, pride, and challenges; and absorption assesses happiness at work. For each domain, participants recorded their feelings about work on a 7-point Likert scale (0=Never, 6=Everyday). Scores for the three statements specific to each of the work engagement dimensions were summed with a possible score ranging from zero to 18. A single score for employee engagement was calculated by averaging the scores of the three statements specific to each of the work engagement dimensions. A higher score indicates greater work engagement.

Job satisfaction: we measured the participants’ job satisfaction using the Job Satisfaction Scale, a validated and reliable research tool.^12^ This scale utilizes four statements in each of nine domains to assess employee attitudes about their job and specific components including pay, promotion, supervision, fringe benefits, contingent rewards, operating procedures, co-workers, nature of work, and communication.^12^ The pay domain addresses perception of pay and remuneration for work; the promotion domain assesses perceptions of promotion opportunities; the fringe benefits domain addresses perceptions of supplemental monetary and nonmonetary benefits for work, and the contingent rewards domain assesses perception of appreciation of a job well done.

**Table 2:** Results for pairwise comparisons using the Holm sequential Bonferroni method

Comparison	Pearson c^2^	p value (α)	Cramer *V*
Dissatisfaction vs ambivalence	10.98	.001 (0.050)^*^	0.23
Ambivalent vs satisfaction	58.91	.0001 (0.017)^*^	0.41
Dissatisfaction vs satisfaction	63.64	.0001 (0.025)^*^	0.58

*p-value ≤ α

The operating procedure domain measures perception of operating policies and procedures of work and the nature of the work domain addresses the perception of job tasks. The supervision domain addresses perceptions of the immediate supervisor; the coworkers' domain measures perception of people at work; and the communication domain addresses the perception of how goals, work assignments and other organizational information are communicated.

For each domain, respondents rated how much a statement applied to them using a 6-point Likert scale (1 = Disagree very much, 6 = Agree very much). The scores from all domains were summed into a job satisfaction score that ranged from 36 to 216, with higher scores indicating high job satisfaction. Job satisfaction scores were categorized into dissatisfaction (<108), ambivalence (108-144), and satisfaction (>144).^2^^,^^14^^,^^27^

Turnover intentions: we measured participants’ turnover intentions using the Boshoff and Allen’s (2000)^28^ 3-item scale. This scale has a high internal consistency coefficient of 0.90. Each item was assessed on a 5-point Likert scale (1 = Strongly disagree, 5 = Strongly agree) and a composite score was calculated by summing the item scores. A higher score corresponds to greater intentions to leave the position. We added the question, “During the past month, have you thought about resigning from this job/position?” to assess intention to resign from the current position with a binary response (Yes or No).

### Data collection

Each FMR manager received an email invitation to participate in the study along with a link to the 59-item survey. We later sent two reminders to those who had not completed the survey. Participation was voluntary, and responses were anonymous. The survey was sent to the 551 FMR managers who were members of AFMA between February and April 2019.

**Table 3:** Results from multiple regression with work engagement as dependent variable

Predictors	Mean	SD	B	SE	β	t	Sig.	F	R	R^2^
(Constant)			0.28	0.27		1.04	0.302	51.05^*^	0.75	0.56
Pay	12.38	5.19	0.02	0.01	0.084	1.85	0.065			
Promotion	10.91	4.09	0.03	0.01	0.112	2.48^***^	0.014			
Supervision	19.47	4.75	0.01	0.01	0.052	1.16	0.247			
Fringe Benefits	16.31	4.52	0.06	0.01	0.284	6.89^*^	0.000			
Contingent Rewards	14.93	4.55	0.01	0.01	0.051	0.96	0.340			
Operating Conditions	12.74	3.22	0.01	0.01	0.043	1.10	0.270			
Coworkers	18.66	3.55	0.02	0.01	0.072	1.53	0.128			
Nature of Work	18.35	3.00	0.22	0.02	0.671	15.06^*^	0.000			
Communication	15.66	4.30	0.03	0.01	0.124	2.57^***^	0.011			

*p < .001; **p < .01; ***p < .05

**Table 4:** Results of hierarchical regression analysis with turnover intentions as dependent variable

	Model 1						Model 2						Model 3				
Predictors	B	SE	β	t	Sig.		B	SE	β	t	Sig.		B	SE	β	t	Sig.
(Constant)	2.84	4.49		0.63	0.528		20.23	3.29		6.15	0.000		20.14	3.21		6.28	0.000
Sex	-0.19	1.56	-0.01	-0.12	0.904		1.28	1.07	0.05	1.20	0.230		1.29	1.04	0.05	1.24	0.216
Employment status	1.54	1.59	0.05	0.97	0.333		0.22	1.08	0.01	0.20	0.838		-0.08	1.05	0.00	-0.08	0.941
Tenure	0.01	0.14	0.01	0.09	0.927		-0.10	0.10	-0.04	-0.96	0.339		-0.06	0.10	-0.03	-0.64	0.523
Annual salary	0.01	0.18	0.00	0.03	0.973		0.13	0.13	0.04	1.02	0.308		0.17	0.13	0.05	1.31	0.192
Highest level of education	0.53	0.17	0.18	3.15^**^	0.002		0.27	0.12	0.09	2.33^***^	0.020		0.28	0.11	0.10	2.51^***^	0.013
Pay							-0.14	0.04	-0.20	-4.03*	0.000		-0.13	0.04	-0.18	-3.71^*^	0.000
Promotion							-0.07	0.04	-0.08	-1.61	0.108		-0.05	0.04	-0.05	-1.14	0.257
Supervision							-0.12	0.04	-0.14	-3.09^**^	0.002		-0.13	0.04	-0.16	-3.51^**^	0.001
Fringe Benefits							0.06	0.04	0.07	1.63	0.104		0.00	0.04	0.00	0.08	0.939
Contingent Rewards							-0.13	0.05	-0.15	-2.65^**^	0.009		-0.11	0.05	-0.13	-2.39^***^	0.017
Operating Conditions							-0.08	0.05	-0.07	-1.60	0.111		-0.09	0.05	-0.07	-1.74	0.082
Coworkers							-0.08	0.05	-0.07	-1.49	0.138		-0.06	0.05	-0.05	-1.11	0.267
Nature of Work							-0.37	0.06	-0.28	-6.11^*^	0.000		-0.16	0.08	-0.12	-2.16^***^	0.032
Communication							-0.12	0.04	-0.13	-2.66^*^	0.008		-0.09	0.04	-0.11	-2.15^***^	0.032
Work engagement													-0.89	0.21	-0.23	-4.31^*^	0.000
R	0.185						0.758						0.773				
R^2^	0.034						0.574						0.597				
ΔR^2^	0.034						0.54						0.023				
F	2.34^***^						31.01^*^						31.75^*^				
ΔF	2.34^***^						45.374^*^						18.53^*^				

*p < .001; **p < .01; ***p < .05

### Statistical analyses

Descriptive statistics were used to examine the variables and to create a demographic profile of respondents. Differences in job satisfaction, turnover intentions, and gender (male vs female) were assessed using Likelihood Ratio Chi-Square tests. Associations among variables were evaluated with Pearson’s correlation. Multiple regression analysis using job satisfaction dimensions was performed to determine the best predictors of work engagement.

We performed a 2-way contingency table analysis to evaluate associations between the categorical turnover intentions classifications and job satisfaction categories.

Hierarchical regression analyses were calculated to identify variables associated with turnover intentions. In the modelling process, we included the independent variables of participant characteristics (sex, employment status, tenure, annual salary, and educational status), job satisfaction domains, and work engagement. All analyses were performed with a two-sided alpha of 0.05 using IBM SPSS (Statistical Package for the Social Sciences) package, version 23.

## Results

### Satisfaction and turnover intentions

**Figure 1:**
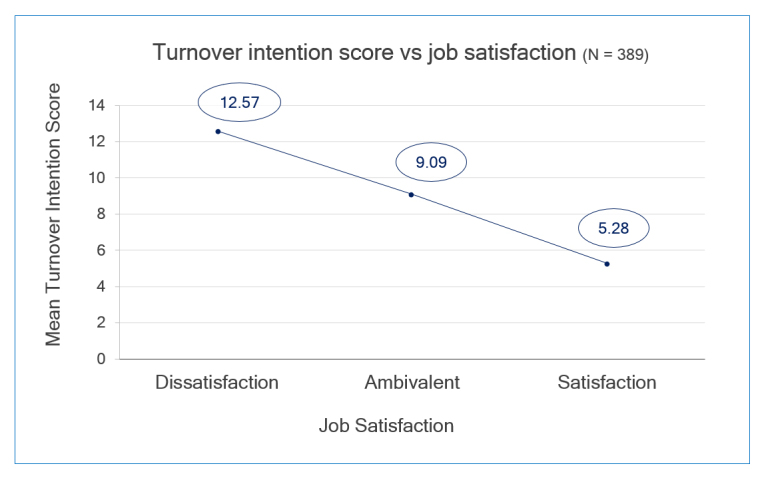
Turnover score and job satisfaction

Of the 551 eligible FMR managers, 389 responded to the survey for a response rate of 70.6%. Forty (10.3% of 389) respondents reported dissatisfaction, 198 (50.9% of 389) ambivalence, and 151 (38.8% of 389) satisfaction about their current work. The mean job satisfaction score was 138.4 (SD = 24.5). Half (194 of 389) had thought about resigning during the past month. The mean score of turnover intentions was 8.0 (SD = 3.8). The mean turnover intentions score declined linearly from 12.58 (standard error [SE] = 0.35) for managers who reported dissatisfaction with their work to 5.28 (SE = 0.23) for those who were satisfied with their jobs (p<0.001), Figure 1.

A 2-way contingency table analysis showed a significant association between job satisfaction and turnover intentions(χ^2^_[2, n = 389]_ = 87.10; p< .001; Cramer V = 0.47). The proportion of job dissatisfaction, ambivalence, and satisfaction were .90, .63, and .22 respectively. Follow-up pairwise comparisons using the Holm sequential Bonferroni method to control for Type I error showed that dissatisfied managers were 4 (.90 vs .22; p < .001) times more likely to report turnover intensions than those who reported satisfaction with their jobs (Table 2).

### Satisfaction, turnover intentions and engagement

Pearson’s correlations were conducted to test hypothesis 1. Work engagement positively correlated with job satisfaction (r_[__387]_ =  .513, p < .001; 2-tailed) and negatively correlated with turnover intentions  (r_[368]_ = - .580, p < .001; 2-tailed). Turnover intentions negatively correlated with job satisfaction (r_[__387]_ = - .690, p< .001; 2-tailed).

We performed multiple regression analysis to test hypothesis 2. The analysis demonstrated that 56% of the variance was explained by the model. Nature of work (t_[364]_ = 15.06, p<.001), fringe benefits (t_[364]_ = 6.89, p<.001), communication (t_[364]_ = 2.27, p<.05), and promotion (t_[364] _= 2.48, p < .05) were significant predictors of work engagement (Table 3).

Table 4 summarizes the regression coefficients, standard error (SE) of the coefficients, standardized beta coefficients (β), t-values, and p-values of the variables in each step used to test hypothesis 3. Of the demographic variables, only educational level was a significant predictor of turnover intentions (t_[__364]_ = 2.51, p < .05). Hierarchal regression analysis further showed that work engagement (t_[364]_ =-4.31, p<.001), pay (t_[364]_ = -3.71, p < .001), supervision (t_[364] _= -3.51, p< .01), contingent rewards (t_[364] _= -2.39, p<.05), nature of work (t_[364]_ = -2.16, p<.05), and communication (t_[364]_ = -2.15, p<.05) were significant, with the model explaining 54% of the variance in turnover intentions (ΔR2 = .54).

## Discussion

This is the first study to provide information regarding job satisfaction, work engagement, and turnover intentions among FMR managers. The findings show that less than 40% are satisfied with their positions and nearly half have thought about resigning within the past month. These findings in crucial members of the residency team are particularly worrying as turnover intentions are predictors of actual turnover.^20^^-^^22^  In addition, about 55% of the managers have held that position for less than five years, confirming other reports that showed similar or greater rates of short tenure among medical residency program managers.^2^^,^^13^^,^^29^ The underlying causes for short tenure and high turnover intentions among medical residency managers are not well defined, but may be related to the combined effects of the expanded job role and responsibilities,^1^^-^^3^ increased job stress without adequate support,^2^ and job dissatisfaction.^21^^,^^23^^,^^24^

Work engagement is positively associated with job satisfaction and negatively associated with turnover intentions.^6^^,^^8^ These findings support and enhance work engagement studies of other employees in diverse industries.^6^^,^^8^^,^^30^ Our findings are in line with studies showing that when employees are satisfied, they are less likely to seek other jobs.^21^^,^^23^^,^^24^^,^^31^ In our study, dissatisfied managers were four times more likely to report turnover intentions.

Of the nine domains of job satisfaction, nature of work, fringe benefits, communication, and promotion significantly predicted FMR manager work engagement. This finding indicates that medical residency managers are more emotionally and cognitively engaged when their job responsibilities are clearly communicated, they have the resources necessary to accomplish their work, perceive their work as meaningful, are compensated fairly and equitably, and have opportunities to be promoted. These findings correlate with engagement findings from studies in other work environments and studies of medical residents that associated job satisfaction positively with work engagement.^10^^,^^11^^,^^31^^,^^32^ Overall, employees are more engaged when they work in an environment that provides practical and psychologically meaningful support as well as advancement and promotion opportunities.^33^ To be effective, support, development and advancement opportunities must be clearly communicated to FMR managers.

Our results show that the prospect of promotion is a predictor of work engagement among managers, but opportunities to advance in medical residency programs are often limited. Currently, many organizational structures do not provide opportunities or a clear pathway for managers to undertake professional development or achieve promotion. In a survey of 400,000 U.S. workers, engagement and employment stability were very high when employees perceived promotion to be fair and managed effectively.^34^ To reduce or minimize turnover intentions and promote greater emotional investment among FMR managers, the accrediting bodies (Graduate Medical Education-International [ACGME-I], ACGME, AAFP [American Academy of Family Physicians], ABFM [American Board of Family Medicine]), AFMA and individual medical residency programs internationally should advance on strategies to support and develop medical residency managers. These strategies could include professional development training and opportunities for lateral movement to develop new skills and expand professional knowledge and collegial support networks.

Finally, in studying the impact of personal characteristics on turnover intentions, level of education significantly predicted turnover intention while, five of the nine job satisfaction domains (pay, supervision, contingent rewards, nature of work, and communication) as well as work engagement significantly predicted turnover intentions with negative coefficients. These findings indicate that FMR managers are more likely to resign when they are highly educated, perceive their pay to be small and unfair, perceive supervisors to be unfair and unsupportive, feel their work is meaningless, job responsibilities are unclear, and perceive they are underappreciated, or are emotionally and cognitively disengaged from work. These findings correlate with multiple studies of employees in diverse work environments.^6^^,^^8^^,^^18^^,^^19^ Clearly, when medical residency managers are emotionally and cognitively engaged at work, they tend to remain in the organization, validating and rewarding organizations that foster employee engagement.^31^^,^^35^

Our study has several limitations. The results are limited to those FMR managers who were members of AFMA at the time of the study and chose to respond to the survey. Although the majority of FMR managers are members of AFMA, responses of non-members could have changed the results of the study. The survey also presents a snapshot of the managers’ subjective responses. As this is a cross-sectional study, we could not establish causal relationship between work engagement, job satisfaction, and turnover intentions; nor can we know whether one preceded the other. Additional research is warranted. Finally, the study was conducted at a time of the year when workload was unusually heavy due to medical residency graduations, orientation of interns, and advancement of resident classes.

## Conclusions

Our study reports high prevalence of job dissatisfaction and turnover intentions among FMR managers and confirms the associations between work engagement, job satisfaction, and turnover intentions. Recognizing the high turnover rates and low job satisfaction among FMR managers should stimulate development of increased opportunities for creative and sustainable solutions. Given the role and level of importance of FMR managers within graduate medical education, our study highlights the importance of developing conversations to improve job satisfaction and work engagement among the managers. Accrediting bodies such as ACGME, AAFP, ABFM, and ACGME-I as well as AFMA need to work with graduate medical education programs to prioritize understanding the needs of FMR managers and implement appropriate programs to support these essential colleagues.

### Acknowledgments

The authors wish to thank Anne Walling, MB ChB for reviewing the manuscript.

### Conflict of Interest

The authors declare that they have no conflicts of interest.

## References

[1] Fountain D, Quach C, Norton D, White SA, Ratliff S, Molteg K, Heyduk D, Roof J, Badurina L (2017). The The perfect storm is on the horizon!. J Surg Educ.

[2] Ofei-Dodoo S, Scripter C, Kellerman R, Haynes C, Marquise ME, Bachman CS (2018). Burnout and job satisfaction among family medicine residency coordinators:results from a national survey.. Fam Med.

[3] Nickel BL, Roof J, Dolejs S, Choi JN, Torbeck L (2018). Identifying managerial roles of general surgery coordinators: making the case for utilization of a standardized job description framework.. J Surg Educ.

[4] Schaufeli WB, Bakker AB, Salanova M (2006). The Measurement of Work Engagement With a Short Questionnaire.. Educational and Psychological Measurement.

[5] Gillet N, Caesens G, Morin AJS, Stinglhamber F (2019). Complementary variable- and person-centred approaches to the dimensionality of work engagement: a longitudinal investigation.. European Journal of Work and Organizational Psychology.

[6] Sibiya M, Buitendach JH, Kanengoni H, Bobat S (2014). The prediction of turnover intention by means of employee engagement and demographic variables in a telecommunications organisation.. Journal of Psychology in Africa.

[7] Soane E, Shantz A, Alfes K, Truss C, Rees C, Gatenby M (2013). The association of meaningfulness, well-being, and engagement with absenteeism: a moderated mediation model.. Human Resource Management.

[8] Coffman C, Gonzalez-Molina G. Gopal A. Follow this path: how the world's greatest organizations drive growth by unleashing human potential. New York: Warner Books, 2002.

[9] Antecedents and consequences of employee engagement: Empirical study of hotel employees and managers [Doctoral Thesis]. Manhattan: Kansas State Universi-ty; 2012. [Cited 02 February 2020]; Available from: http://hdl.handle.net/2097/13653.

[10] Agarwal G, Karpouzian T (2016). An exploratory analysis of work engagement, satisfaction, and depression in psychiatry residents.. Acad Psychiatry.

[11] Zis P, Anagnostopoulos F, Artemiadis AK (2016). Residency training: work engagement during neurology training.. Neurology.

[12] Spector PE (1985). Measurement of human service staff satisfaction: development of the job satisfaction survey.. Am J Community Psychol.

[13] Ofei-Dodoo S, Irwin G, Kuhlmann Z, Kellerman R, Wright-Haviland S, Dreiling M (2019). Assessing work-related burnout and job satisfaction among Obstetrics and Gynecology residency program coordinators.. Kans J Med.

[14] Ofei-Dodoo S, Scripter C, Kellerman R (2018). Job satisfaction and burnout among nonclinical workers in a medical education center.. Fam Med.

[15] Lu ACC, Gursoy D (2016). Impact of job burnout on satisfaction and turnover intention.. Journal of Hospitality and Tourism Research.

[16] Bakker AB, Demerouti E, Sanz-Vergel AI (2014). Burnout and work engagement: the JD-R approach.. Annual Review of Organizational Psychology and Organizational Behavior.

[17] Qureshi MI, Iftikhar M, Abbas SG, Hassan U, Khan K, Zaman K. Relationship between job stress, workload, environment and employees turnover intentions: what we know, what Should we know? World Applied Sciences Journal. 2013;23(6):764-770.

[18] Ongori H. A review of the literature on employee turnover. African Journal of Business Management. 2007;1(2):49-54.

[19] Firth L, Mellor DJ, Moore KA, Loquet C (2004). How can managers reduce employee intention to quit?. Journal of Managerial Psychology.

[20] van Breukelen W, van der Vlist R, Steensma H (2004). Voluntary employee turnover: combining variables from the 'traditional' turnover literature with the theory of planned behavior.. J Organiz Behav.

[21] Saeed I, Waseem M, Sikander S, Rizwan M. The relationship of turnover intention with job satisfaction, job performance, leader member exchange, emotional intelligence and organizational commitment. International Journal of Learning and Development. 2014;4(2):242-256.

[22] O’Connor J (2018). The impact of job satisfaction on the turnover intent of executive level central office administrators in Texas public school districts: a quantitative study of work related constructs.. Educ Sci.

[23] Larkin I, Brantley-Dias L, Lokey-Vega A. Job satisfaction, organizational commitment, and turnover intention of online teachers in the K-12 setting. Online Learning. 2016;20(3):26-52.

[24] Egan TM, Yang B, Bartlett KR (2004). The effects of organizational learning culture and job satisfaction on motivation to transfer learning and turnover intention.. Human Resource Development Quarterly.

[25] Scanlan JN, Still M (2019). Relationships between burnout, turnover intention, job satisfaction, job demands and job resources for mental health personnel in an Australian mental health service.. BMC Health Serv Res.

[26] Australia and New Zealand melanoma trials group statistical decision tree. power calculation for Pearson's and spearman's correlation. [Cited 12 March 2019]; Available from: https://www.anzmtg.org/stats/PowerCalculator/PowerTtest.

[27] Spector PE. Job satisfaction survey: interpreting satisfaction scores with the job satisfaction survey. [Cited 13 March 2019]; Available from: http://shell.cas.usf.edu/~pspector/scales/jssinterpretation.html.

[28] Boshoff C, Allen J (2000). The influence of selected antecedents on frontline staff's perceptions of service recovery performance.. International Journal of Service Industry Management.

[29] Ewen AM, Gardiner PM, Palma S, Whitley K, Schneider JI (2018). We matter too! addressing the wellness of program coordinators in graduate medical education.. J Contin Educ Health Prof.

[30] May DR, Gilson RL, Harter LM (2004). The psychological conditions of meaningfulness, safety and availability and the engagement of the human spirit at work.. Journal of Occupational and Organisational Pscychology.

[31] MacIntosh EW, Doherty A (2010). The influence of organizational culture on job satisfaction and intention to leave.. Sport Management Review.

[32] Lu L, Lu ACC, Gursoy D, Neale NR (2016). Work engagement, job satisfaction, and turnover intentions.. International Journal of Contemporary Hospitality Managemen.

[33] Kahn WA (1990). Psychological conditions of personal engagement and disengagement at work.. AMJ.

[34] Rohman J, Onyeagoro C, Bush MC. How you promote people can make or break company culture. Harvard Business Review. Updated January 2, 2018. [Cited 2 July 2019]; Available from: https://hbr.org/2018/01/how-you-promote-people-can-make-or-break-company-culture.

[35] Bakker AB, Schaufeli WB (2008). Positive organizational behavior: engaged employees in flourishing organizations.. J Organiz Behav.

